# Costimulation Blockade in Vascularized Composite Allotransplantation

**DOI:** 10.3389/fimmu.2020.544186

**Published:** 2020-09-17

**Authors:** Dimitrios Giannis, Dimitrios Moris, Linda C. Cendales

**Affiliations:** ^1^Institute of Health Innovations and Outcomes Research, Feinstein Institutes for Medical Research, Manhasset, NY, United States; ^2^Duke Surgery, Duke University Medical Center, Durham, NC, United States

**Keywords:** vascularized composite allotransplantation, belatacept, costimulation blockade, VCA, hand transplantation

## Abstract

Vascular composite allotransplantation (VCA) is a field under research and has emerged as an alternative option for the repair of severe disfiguring defects that result from infections or traumatic amputation in a selected group of patients. VCA is performed in centers with appropriate expertise, experience and adequate resources to effectively manage the complexity and complications of this treatment. Lifelong immunosuppressive therapy, immunosuppression associated complications, and the effects of the host immune response in the graft are major concerns in VCA. VCA is considered a quality of life transplant and the risk-benefit ratio is dissimilar to life saving transplants. Belatacept seems a promising drug that prolongs patient and graft survival in kidney transplantation and it could also be an alternative approach to VCA immunosuppression. In this review, we are summarizing current literature about the role of costimulation blockade, with a focus on belatacept in VCA.

## Introduction

Vascular composite allotransplantation (VCA) is a field under research and has emerged as an alternative option for the repair of severe disfiguring defects that result from infections or traumatic amputation in a selected group of patients (1). VCA consist of anatomically distinct tissues such as skin, muscles, connective tissue stroma, bones and neurovascular elements that are transplanted as functional complexes (2–5). It can be applied to various body parts, such as the face, upper or lower extremity, the larynx, the abdominal wall, as well as intra-abdominal organs, such as the spleen, the adrenals or genitourinary organs (5–7).

The first attempt for a human hand transplantation was done in 1964 in Ecuador, but acute rejection (AR) led to graft amputation at 3 weeks postoperatively, despite the use of combined immunosuppression therapy with azathioprine and prednisone (8) (Table 1). Many years have passed since then, until another VCA transplantation was performed. Newer immunosuppressive drugs emergenced, such as cyclosporine and tacrolimus, combined with improved solid organ transplantation (SOT) and non-human primates (NHP) VCA outcomes have led to a new era in human VCA transplantation (9–15). The first technically successful forearm transplantation was performed in 1998 in Lyon, France, but the initially viable graft was eventually rejected and amputated due to patient non-compliance with immunosuppressive treatment, consisting of tacrolimus, mycophenolic acid and prednisone (16, 17). Hand transplantation in the United States was firstly performed in Louisville, Kentucky in 1999 with functional improvement compared to prosthesis and allograft currently surviving 20 years post-transplantation (9, 18). Hand transplantation is currently the most common VCA performed clinically.

**Table 1:** Important milestones of VCA since its introduction to the transplant community.

**Year**	**Milestone**
1964	First attempt of clinical vascularized composite allotransplantation (VCA)
1998	First succesful VCA
2000	First Uterine Transplantation
2007	Banff Classification of VCA clinical rejection
2015	Belatacept in human VCA

In 2005, the first face transplantation was reported by the Amiens transplantation team in a woman suffering from traumatic amputation of the lower face, including distal nose, lips, chin, and lateral face parts (19). Since then at least 46 facial transplantations have been performed worldwide in patients with burn injuries, animal bites or malignancies (20–23). Face transplantation re-establishes the ability of patients to speak, feed themselves and express their emotional status, thus facilitating an adequate social life (6). Immunosuppression regimens are similar to the scheme used in hand transplantations, consisting of induction therapy, such as anti-thymocyte globulin (ATG), alemtuzumab or basiliximab followed by the standard triple drug regimen, which includes tacrolimus, mycophenolate mofetil (MMF) and steroids (6, 24–26).

Advances in the field of VCA led to the first time uterine transplantation (UTx). It was first attempted by Fageeh et al. in Saudi Arabia in 2000 on a 26 year-old female in the context of previously performed hysterectomy due to post-partum hemorrhage. The uterus graft underwent acute thrombosis 3 months post-transplantation and eventually hysterectomy was performed (27). The first successful pregnancy and livebirth after UTx was achieved in Gothenburg, Sweden in 2015, demonstrating that UTx may be a feasible fertility-restoring option for women with uterine factor infertility (28). The Swedish group has paved the way in the establishment of UTx as a viable option for infertility and are leaders in uterus tissue engineering (29) and minimally invasive UTx (30, 31). Ejzenberg et al. recently reported the first successful livebirth from deceased donor in Brazil in a young patient with Mayer-Rokitansky-Küster-Hauser syndrome (32). In the United States, the field is rapidly growing. In 2017, the first 5 cases of UTx from living donors were reported (33). In 2018, the same group reported the first livebirth after UTx from altruistic living donor (34). To date, more than 60 UTx have been performed globally and 18 offspring have been reported to have been successfully delivered (35).

The main limitation of currently used immunosuppression regimens are side effects. Specifically, tacrolimus, which is a calcineurin inhibitor (CNI) is a core component of maintenance regimens in VCA worldwide and has been reported as one of the main causes of complications in VCA. Barth et al. reviewed the VCA experience and identified 4 cases (3 limb and 1 face) with renal failure or progressive renal dysfunction up to 8 years post-transplantation. One patient has been transplanted and 3 are listed for renal transplantation (36). Alternative agents have emerged in an effort to minimize or replace the use of tacrolimus and associated side effects. Belatacept-based immunosuppression in preclinical VCA models and clinical VCA has recently emerged as a promising alternative treatment to counteract long-term adverse effects of currently used chronic immunosuppression agents (25, 37–39). Belatacept is a fusion protein (CTLA4-IgG1) that targets the CD28/B7 costimulation between T- and B-lymphocytes. The BENEFIT phase 3 randomized controlled study revealed increased patient and graft survival as well as improved renal function with belatacept at seven years post-transplantation compared to cyclosporine-based regimen (40). Furthermore, the risk of death or long-term graft loss reduced by 43% with both belatacept regimens tested and the glomerular filtration rate (GFR) increased compared to cyclosporine-nephrotoxicity GFR decrease (40). In this review, we are focusing on summarizing current literature about the role of costimulation blockade in VCA.

## VCA and Rejection

The increasing numbers of VCA along with advances in immunomodulation schemes mandate the need of a universally accepted histological classification. The Banff 2007 classification was a milestone in the characterization and appropriate reporting of VCA rejection. The skin as a visible component of the transplant provides an easily and accessible monitored graft area facilitating AR recognition. According to Banff 2007 the skin rejection severity is classified in five grades ranging between 0 and IV (41). AR manifests with skin lesions, such as macules, papules, erythema, edema and nail changes (42, 43). AR initially involves neutrophils and T-cells producing chemoattractive factors acting on macrophages (IFN-γ) (Table 2). If not reversed, AR progresses from mild perivascular dermis inflammation to epidermal and adnexal cellular infiltration and eventually irreversible epidermal necrosis (41, 44). Repeated episodes of AR are considered important contributors to chronic allograft dysfunction. Multiple AR episodes result in persistent chemokine elevation and macrophage graft infiltration, resulting in fibroblast proliferation and collagen deposition through macrophage secreted cytokines (FGFβ, TGFβ and PDGF) (45, 46). Histopathology of chronic graft functional deterioration involves myointimal proliferation, fibrosis, vasculopathy and parenchymal structural dysregulation (45, 47). Despite initial beliefs underestimating the role of antibodies in VCA graft damage, it is currently established that antibody-mediated rejection is also an important process affecting graft viability (48, 49). The vascular component of the graft is a target in chronic rejection and by donor specific antibodies (DSA), which may develop years after transplantation and have been associated with CNI immunosuppression sparing regimens (50, 51).

**Table 2:** Rejection mechanisms involved in VCA.

**Rejection classification**	**Primary findings**
**Type of rejection**	
Acute Rejection	Endothelial injury, dermal perivascular CD4/CD8 infiltrates
Chronic Rejection	Microthromboses, graft vasculopathy
**Immunologic mechanism**	
Cell-Mediated	CD4/CD8, adhesion molecules, pro-inflammatory cytokines
Antibody-Mediated	B cells, C4d deposits, DSA

*DSA, Donor specific antibodies*.

## VCA Immunosupression: Conventional Immunosuppressive Drugs

Currently used immunosuppressive drug regimens protect from early graft loss but are unable to prevent rejection in up to 90% of all VCA recipients and are associated with serious adverse effects (6) (Table 3). Opportunistic infections due to viruses, such as EBV, CMV, HSV-1, have been extensively reported and investigated in SOT recipients and have been identified as major complications affecting VCA patients (6, 70–72). Cancer commonly affects transplant recipients and is an emerging issue in VCA transplantation, in the form of *de novo* tumorigenesis, associated with immunosuppression *per se* or viral reactivation (EBV related lymphomas), or tumor recurrence (20, 73). The most commonly used induction agent in VCA is antithymocytic globulin (ATG) and acts through T-cell depletion as a polyclonal antibody directed against the CD2, CD3, CD4, and CD8 molecules. ATG induction results in decreased T-cell mediated rejection, which is an common observation in VCA rejection (74–76). ATG side effects include leukopenia, thrombocytopenia, serum sickness, cytokine release syndrome, and infections (55, 56). Corticosteroids are considered as a milestone of transplantation immunosuppressive therapy. Nevertheless, their side-effects, such as myopathy, diabetes mellitus, hyperlipidemia, osteoporotic fractures, impaired wound healing, have led to the emergence of steroid sparing regimens with promising results in SOT (61–63). Tacrolimus and cyclosporine are calcineurin inhibitors and their well-known detrimental effects include impaired kidney function (acute and chronic nephrotoxicity), glucose metabolism (hyperglycemia) and lipid metabolism (dyslipidemia) (64–66). Tacrolimus to sirolimus (mTOR kinase inhibitor) conversion has been successfully used in VCA in order to counteract renal toxicity (77). Mycophenolate Mofetil (MMF), commonly used as maintenance drug, acts as inosine monophosphate dehydrogenase (IMPDH) inhibitor and interferes with *de novo* purine nucleotide synthesis, which is essential for the proliferation of lymphocytes (78). Main adverse reactions associated with MMF include abdominal pain, vomiting, leukocytopenia and diarrhea (63).

**Table 3:** Mechanisms and adverse effects of currently used immunosuppression drugs in VCA.

**Drug**	**Mechanism**	**Timing**	**Main adverse effects**
**IMMUNOSUPPRESSION IN VCA**			
Alemtuzumab	CD52 mAB	Induction	Hypersensitivity reaction, anemia, neutropenia, thrombocytopenia (52–54)
Antithymocyte globulin (ATG)	T-cell depletion (polyclonal antibody against CD2,CD3,CD4,CD8)	Induction	Leukopenia, thrombocytopenia, serum sickness, cytokine release syndrome (55, 56)
Basiliximab	IL-2R mAB	Induction	Hypersensitivity reaction (mild or anaphylaxis) (57–59)
Belatacept	CD28—B7 blockade	Maintenance	Post-transplantation lymphoproliferative disorder (PTLD) (40, 60)
Corticosteroids	Lymphocytolysis	Maintenance	Infection, myopathy, DM, hyperlipidemia, osteoporosis (61–63)
Cyclosporine	Calcineurin inhibitor	Maintenance	Nephrotoxicity, DM, dyslipidemia (64–66)
MMF	IMPDH inhibitor	Maintenance	Abdominal pain, vomiting, leukocytopenia, diarrhea (63)
Sirolimus, Everolimus	mTOR Inhibitor	Maintenance	Hypertriglyceridemia, mouth ulcers, leukopenia, anemia, thrombocytopenia, impaired wound healing, drug-induced pneumonitis (67–69)
Tacrolimus	Calcineurin inhibitor	Maintenance	Nephrotoxicity, DM, dyslipidemia (64–66)

## VCA Immunosuppresion: Monoclonal Antibodies

The optimal immunosuppressive regimen would prevent rejection as well as have minimal or no major toxicity over a prolonged period of use. Monoclonal antibodies (mAbs) have been tested as promising agents in VCA immunosuppressive regimens.

## Alemtuzumab and Basiliximab

Alemtuzumab, a humanized mAb targeting CD52 (GPI-linked surface protein of mature lymphocytes) that causes B and T lymphocyte depletion, has been used in the prevention of SOT AR as well as in the treatment of VCA AR episodes (24, 79, 80). Basiliximab, a chimeric mAb specifically binding to interleukin-2 receptor, has been tested with promising results in SOT recipients and has also been used as induction therapy in VCA recipients (26, 81–83).

## Belatacept: Mechanism, Benefits and Side Effects

The development of belatacept along with its prototype molecule CTLA4-Ig introduced a new class of immunosuppressive agents called costimulation blockade agents (84). T-cells become activated in the presence of three signals: One signal is mediated through the interaction of T-cell receptor (TCR) and major histocompatibility complex (MHC) molecules, an additional accessory costimulatory signal is mediated through the interaction of other cell surface molecules and the third signal is delivered in the form of cytokines (85). Naïve helper (CD4+) and cytotoxic (CD8+) T-cells must undergo activation in order to become effectors cells able to participate in graft rejection (86). Belatacept is a fusion protein consisting of cytotoxic T-lymphocyte–associated antigen 4 (CTLA-4 or CD152) and IgG1 Fc portion acting through the blockade of CD28 (T-cell surface) and B7 (B-cell surface) costimulation. Belatacept was approved as a CNI replacement therapy in kidney transplant recipients with significantly better renal function, patient and graft survival compared to cyclosporine (40, 87). However, the BENEFIT study demonstrated high cumulative rates of acute rejection in both high (24.4%) and low (18.3%) intensity belatacept groups compared to cyclosporine (11.4%) at 7 years post-transplantation. Higher rate of donor specific DSA formation was observed in cyclosporine treated patients (17.8%) compared to the high intensity (1.9%) and low intensity (4.6%) belatacept regimens (40). Belatacept is more effective in the prevention of the development of DSA, but may be inferior to CNIs in the short term in the prevention of acute cellular rejection. Safety profile is comparable, if not better, to CNI drugs (88). Specifically, belatacept offers better blood pressure management, lipid metabolic profile and lower incidence of post-transplantation diabetes (89, 90). In addition, there is no difference in risk of developing cancer or infectious complications in kidney transplantation between belatacept and CNI (90). Belatacept treatment is limited to EBV-seropositive recipients, because some seronegative patients of the BENEFIT study, who were given higher doses, developed post-transplantation lymphoproliferative disorder (PTLD) (40, 60). In addition, belatacept is not indicated in liver transplantation based on a phase II study report that associated belatacept with increased graft loss and mortality compared to MMF + tacrolimus (91).

## CTLA4-Ig and Belatacept in VCA Experimental Models

Costimulation immunosuppression with CTLA4-Ig has been shown to prolong survival of donor skin grafts in rat cardiac allograft transplantation compared to third-party skin grafts, indicating donor-specific tolerance induction (92) (Table 4). Combination of CD28 and CD40 pathway blockade promotes survival of allogeneic skin grafts in the context of T-cell clonal expansion prevention (96). Iwasaki et al. used a rat hind limb VCA model to demonstrate that CTLA4-Ig regimen on day 2 significantly prolongs graft survival compared to vehicle and control groups (hIgG administration and no treatment, respectively) (Median graft survival: 20.5, 9, and 9 days, respectively, *p* < 0.01) and all CTLA4-Ig treated histologic specimens remained unaffected at 7 days post-transplantation (95). In addition, the same study showed that CTLA4-Ig optimally inhibits allograft rejection when administered on postoperative days 1 or 2 compared to immediate post-transplant treatment (95). Foster et al., using a model consisting of fully mismatched donor and recipient rats, showed that donor bone marrow (BM) administered to recipients, at 4 weeks prior to hind limb VCA transplantation, combined with CTLA4-Ig could effectively prevent acute and chronic rejection of the allograft (94). VCA hind limb allograft survival in swines has been shown to benefit significantly by CTLA4-Ig + Tacrolimus combination compared to Tacrolimus + BM transplantation + Irradiation or Tacrolimus only regimens, with a great impact on skin component rejection prevention (100). Lin et al. utilized a combination of anti-CD154 (anti-CD40L), CTLA4-Ig and rapamycin (RPM) in mice osteomyocutaneous allografts transplantation and reported long-term survival in the anti-CD154 + CTLA4-Ig+RPM group compared to anti-CD154 + CTLA4-Ig or RPM only groups (Median survival time: 103, 33, 45.8 days, respectively) (97). In the aforementioned study, long graft survival was associated with increased number of T-regulatory cells (Tregs) and decreased CD4+ and CD8+ counts (97). More recently, Oh and colleagues tested the combination of CTLA4-Ig + anti-CD154 + total body irradiation in a fully MHC-mismatched mouse hindlimb model and reported a graft survival of over seven months compared to 82 days in the group treated with CTLA4-Ig + anti-CD154 only (98). Lastly, Schweizer et al. used adipose-derived mesenchymal stem cells combined with CTLA4-Ig and antilymphocyte serum in a rat hindlimb model, in addition to tacrolimus, and achieved an over 4 months rejection free allograft survival compared to control groups (median graft survival <35 days) (99).

**Table 4:** Summary of studies evaluating the role of costimulation blockade in VCA NHP models.

**References**	**Model**	**Tissue**	**Regimen**	**Graft survival**	**Cell population affected (mechanism)**
**PRECLINICAL STUDIES OF COSTIMULATION BLOCKADE IN VCA**					
Atia et al. (93)	Monkey	Forearm	Belatacept + Steroids (3 groups: With ustekinumab, with secukinumab or without additional drugs)	Decreased (in all groups compared to historical control)	T cells (DSA formation prevention; decreased T cells, IL-17a T cells and IL-17a in ustekinumab and secukinumab treated animals)
Foster et al. (94)	Rat	Hindlimb	CTLA4-Ig + BM	Prolonged	T-cells (clonal expansion inhibition, mixed chimerism)
Freitas et al. (38)	Monkey	Forearm	CTLA4-Ig/Belatacept + Tacrolimus	Prolonged	T-cells (bimodal distribution of CD2^lo^ and CD2^hi^ CD^8+^ T cells, DSA formation prevention)
Iwasaki et al. (95)	Rat	Hindlimb	CTLA4-Ig	Prolonged	T-cells (mixed chimerism)
Larsen et al. (96)	Mouse	Skin	CTLA4-Ig + MR1(CD40 blockade)	Prolonged	T-cells (clonal expansion inhibition)
Lin et al. (92)	Rat	Skin	CTLA4-Ig + DST	Prolonged	Lymphocytes (50% reduced *in vitro* proliferative response)
Lin et al. (97)	Mouse	Hindlimb	CTLA4-Ig + anti-CD154 +RPM	Prolonged	T-cells (Increased Tregs, decreased CD4+, CD8+ counts)
Oh et al. (98)	Mouse	Hindlimb	CTLA4-Ig + anti-CD154 +TBI	Prolonged	T-cells(clonal deletion of donor-reactive T cell clones, mixed chimerism, Increased Tregs)
Schweizer et al. (99)	Rat	Hindlimb	Tacrolimus+CTLA4-Ig+ASC+ALS	Prolonged	T-cells (Increased Tregs, mixed chimerism)
Wachtman et al. (100)	Swine	Hindlimb	CTLA4-Ig+Tacrolimus	Prolonged	NR

*ALS, antilymphocyte serum; ASC, Adipose tissue derived stem cells; BM, Bone marrow; DSA, Donor specific antibodies; DST, Donor-specific cell (splenocyte) transfusion to graft recipient; MR1, Anti-CD40L mAb; NR, Not reported; RPM, Rapamycin TBI, Total body irradiation*.

Preclinical data in VCA have already demonstrated the efficacy of belatacept as a maintenance treatment in VCA (38, 39). Freitas et al. reported that, in a cynomolgus monkey model of forearm VCA, costimulation blockade in the form of CTLA4-Ig or belatacept combined with tacrolimus improved graft survival and prevented DSA formation compared to tacrolimus + steroids (38). Recently, Atia et al. investigated the effect of belatacept in combination with Th17 response inhibitory drugs (ustekinumab and secukinumab) in a rhesus macacques model of VCA. The comparison with a historic cohort, treated with the standard immunosuppression (Tacrolimus, MMF, Methylprednisolone), revealed significantly shorter interval to acute rejection in all groups (≤14 days), independent of the Th17 inhibition, compared to controls (mean survival = 31.1 days). However, historic controls without costimulation blockade showed a significant increase in DSA production at rejection, while all belatacept treated animals did not develop post-transplant DSA at rejection (93).

## Belatacept in Clinical VCA

Belatacept has been successfully used as CNI replacement treatment to counteract CNI-induced nephrotoxicity in a 21 year old female hand transplant recipient with left wrist amputation due to Kawasaki vasculitis affecting the extremities. The patient developed recurrent episodes of acute rejection with alloantibody formation and initial maintenance treatment consisting of tacrolimus, MMF and steroids was replaced by belatacept, sirolimus and steroids at 12 months post-transplantation (25) (Table 5). Belatacept based costimulation blockade was applied based on the diagnostic confirmation of antibody-mediated rejection and previous results of belatacept efficacy on the prevention of alloantibody formation (25, 102). After conversion, at 42 months post-transplantation and 30 months on belatacept, no episodes or signs of rejection occurred in the normally functioning allograft (25).

**Table 5:** The role of Belatacept in VCA clinical practice.

**References**	**Gender, Age**	**VCA**	**Timing**	**Rejection**
Cendales et al. (25)	Female, 21	Unilateral hand	12 months (conversion)	No
Krezdorn et al. (101)	Male, NR	Face	14 months (conversion)	Yes (BRR)
Grahammer et al. (39)	Males (4 pts), NR	2 Bilateral Hand, 1 Bilateral hand & forearm, 1 Unilateral hand	3 months−13 years (conversion)	Yes (2 pts)
Cendales et al. (37)	Male, 54	Unilateral hand	Initial maintenance	Yes

*Pts, patients; BRR, Belatacept resistant rejection*.

Krezdorn et al. investigated the immunologic response of a face transplantation recipient who developed belatacept resistant rejection (BRR) after belatacept conversion in the context of tacrolimus and rapamycin adverse effects (101). Initially, tacrolimus + MMF + prednisone were applied with tacrolimus to rapamycin conversion at 11 months post-transplantation due to impaired renal function and neurotoxicity as well as CMV infection. Subsequently, at 14 months post-transplantation, further deterioration of renal function and cell-mediated rejection led to belatacept conversion. Nevertheless, 4 months post-conversion the patient developed rejection and was additionally treated with low-dose tacrolimus to achieve remission (101). Previous data derived from costimulation-based regimens in kidney transplantation revealed that a specific subset of CD4+ T cells (CD4+CD57+PD1-) with cytolytic properties may act as efficient high-risk marker of BRR (103). Nevertheless, this CD4+ subset was not significantly elevated in this face transplant recipient prior to or after belatacept initiation. Interestingly, both Tregs and Tfh cell counts were decreased during belatacept treatment, whereas Th1 and Th17 counts increased (101). Belatacept-induced Tfh count decrease has been previously shown to suppress humoral immunity and antibody-mediated rejection (104).

Recently, belatacept was tested as maintenance treatment in 4 male hand-transplanted patients with beneficial results and limitations as well. In two DSA-negative bilateral hand recipients, belatacept treatment was not associated with rejection in spite of decreases in tacrolimus dosage (patient 1) or cessation of everolimus administration (patient 2) (39). Patient 3 received bilateral forearm transplantation and had suffered from recurrent cell-mediated rejection and one DSA (+) rejection episode prior to belatacept initiation at 9 years post-transplantation. He remained free of rejection without detection of DSA along with improved graft macroscopic image and function. The last patient received belatacept at 6 years post-transplantation due to CNI caused nephrotoxicity, but at 2 months of costimulation-blockade acute rejection occurred and was treated with alemtuzumab conversion. Eventually, 8 months later resistant rejection led to removal of the transplanted hand. Patient 4 immunologic profile revealed that CD4+CD57+ T-cells were increased compared to long-term graft survival patients (39).

Our group investigated the role of *de-novo* belatacept in VCA (37). A 54 year-old male transplant recipient, suffering from traumatic amputation of the left hand, was treated with belatacept, MMF, steroids and tacrolimus, followed by conversion to sirolimus at 6 months. At 8 months post-transplantation macroscopically (erythematous maculopapular rash) and microscopically confirmed rejection Banff III (41), which was successfully treated with IV steroids. At 20 months post-transplantation the patient was reported to be free of rejection, with improved graft function in daily activities and maintained on belatacept + MMF + prednisone (37). This study demonstrated that belatacept can be incorporated as a core component of antirejection regimens, minimizing the use of CNI and their long-term adverse effects.

## Belatacept in VCA: Advantages and Limitations

Currently, belatacept seems as a promising agent that prolongs the rejection free survival when added to tacrolimus in experimental VCA models (38). However, belatacept in combination with steroids alone failed to prevent acute rejection and resulted in an average rejection free survival (time from transplant to early signs of rejection) of 10 days compared to an average rejection free survival 31.1 days in animals treated with tacrolimus, MMF and steroids (93, 105). As it has been shown by us and others, the use of belatacept resulted in inhibition of DSA formation (39, 93). As anticipated and similar to other organ transplants graft vasculopathy and antibody mediated rejection in VCA are associated with the presence of DSA or C4d deposits (106–110). The incidence of acute rejection in VCA has been reported to 85% within the first year (39, 76), which is higher than other solid organ transplants (111–113). Belatacept is associated with a higher incidence of acute rejection episodes in kidney (40). Considering that VCA has a higher reported incidence of skin changes attributed to rejection, even in MHC-matched VCA transplants (100), this would be an area to study and to report as more cases are performed. An important consideration is differential diagnosis. Specifically, in VCA, the skin is the monitoring tool for rejection in VCA and studies by our VCA collaborative initiative have shown that skin is the harbinger of rejection (114). However, the skin changes in VCA -although characteristic- they are non- specific (115). Similarly, they are not limited to alloimmune injury. Thus, the diagnosis of rejection can be challenging as multiple unrelated inflammatory dermatoses can mimic alloimmune driven acute rejection (e.g., infectious, drug toxicity) (115). As the field continues to develop and more data become available particularly as they relate to differential diagnosis of rejection, the incidence of the skin changes attributed to rejection may change.

Based on studies in swine, CTLA4-Ig and CNIs are effective in preventing allograft rejection. In a study by Wachtman et al. in swine CTLA4-Ig was utilized in combination with tacrolimus (CNI was stopped at 30 days). The CTLA4-Ig regimen resulted in a prolonged survival in animals (indefinite in two animals) while the tacrolimus alone group resulted in rejection 2 days after tacrolimus cessation (100). Our studies on NHPs show the benefit of belatacept in VCA. The addition of *de novo* belatacept to a regimen consisting of tacrolimus, steroids, and conversion to sirolimus, significantly prolonged the rejection free survival (Up to 140 days in belatacept vs. 14 days in non-belatacept regimen) (38). Based on these findings in large animal models and our human clinical trial (NCT02310867), a regimen that deserves ongoing consideration is *de novo* belatacept, calcineurin inhibitors for the initial 6 months to avoid the negative impact of sirolimus on wound healing (38) followed by conversion to sirolimus (25).

A main clinical concern with belatacept treatment is the risk of post-transplantation lymphoproliferative disorder (PTLD) in EBV seronegative transplant recipients (40, 116). In addition and similar to other organ transplants, immunomodulation during conversion requires surveillance for potential increase rejection. As described by Grahammer et al., hand transplant recipients who remained clinically stable prior to and after belatacept conversion, were free of cellular rejection and DSA formation at time of belatacept initiation (39).

Costimulation blockade alone is ineffective in inducing long-term graft allograft survival or tolerance in animal transplant models (98, 117, 118). The inhibition of CD28/B7 costimulation pathway affects various immune cells, including Tregs and Th17 cells, while the time course and regimen intensity are critical predictors of the ultimate response (119). Costimulation pathway CD28/B7 is important for the activation of Tregs, which is important for the induction of tolerance (119, 120). An unfavorable effect of belatacept on Tregs was shown in a face transplant recipient who presented with rejection 4 months after conversion to belatacept (101). It has also been shown that costimulation blockade has a is limited effect on memory T cells, which are less dependent on CD28/B7 activation and have been implicated in belatacept resistant rejection (39, 121–125). Potential co-targeting of these memory cells to bypass this limitation may predispose to increase risk of infections (119). In addition, costimulation blockade with agents targeting the B7 molecule, not only inhibit CD28/B7 interaction, but prevent CTLA4/B7 interaction and programmed death-ligand 1 (PD-L1)/B7 interaction and T-cell co-inhibition. Impairment of T-cell co-inhibition results in ineffective control of alloreactive T-cell activation, including effector memory T cells and Th17 cells (119, 126, 127).

## Summary

VCA is a field under development and is performed in centers with appropriate expertise, experience and adequate resources to effectively manage the complexity and complications of this treatment option. Similar to other solid organ transplants, lifelong immunosuppressive therapy, their complications, and the effects of the alloimmune response in the graft are major concerns in VCA. VCA is a quality of life transplant and the risk-benefit ratio is dissimilar to life saving transplants. Belatacept seems a promising drug that prolongs patient and graft survival in solid organ transplantation and it could also be an alternative approach to VCA immunosuppression. A regimen that deserves ongoing consideration is *de novo* belatacept to avoid chronic CNI exposure.

## Author Contributions

DM and LC: conception or design of the work. DG and DM: data collection. DG, DM, and LC: data analysis and interpretation, drafting the article, critical revision of the article, and final approval of the version to be published. All authors read and approved the final manuscript.

## Conflict of Interest

The authors declare that the research was conducted in the absence of any commercial or financial relationships that could be construed as a potential conflict of interest.
